# Editorial: Neuromuscular Training and Adaptations in Youth Athletes

**DOI:** 10.3389/fphys.2018.01264

**Published:** 2018-09-10

**Authors:** Urs Granacher, Christian Puta, Holger H. W. Gabriel, David G. Behm, Adamantios Arampatzis

**Affiliations:** ^1^Research Focus Cognition Sciences, Division of Training and Movement Sciences, University of Potsdam, Potsdam, Germany; ^2^Department of Sports Medicine and Health Promotion, Friedrich-Schiller-University Jena, Jena, Germany; ^3^School of Human Kinetics and Recreation, Memorial University of Newfoundland, St. John's, NL, Canada; ^4^Department of Training and Movement Sciences, Humboldt-Universität zu Berlin, Berlin, Germany

**Keywords:** strength training, plyometric training, physical fitness, injury prevention, athletic performance

Myer et al. (2011b) defined neuromuscular training (NT) as a training program that incorporates general (e.g., fundamental movements) and specific (e.g., sport-specific movements) strength and conditioning activities, such as resistance, dynamic stability, balance, core strength, plyometric, and agility exercises with the goal to enhance health- and skill-related physical fitness components and to prevent injuries. According to this definition, agility, balance, plyometric, power, stability, and strength training are subsets of NT.

Over the past decades, the number of scientific publications on NT in non-athletic youth grew exponentially and provided convincing evidence to overcome long-term held myths on detrimental effects of particularly strength training in youth (e.g., damage to growth plates, high injury risk) (Figure 1). Today, the positive effects of NT in general and strength training in particular are well-documented. Findings from original work, systematic reviews and meta-analyses proved the effectiveness of NT on muscular fitness, motor skills, sports performance, resistance to injuries, metabolic and mental health in non-athletic youth (Behringer et al., 2011; Myer et al., 2011a; Faigenbaum et al., 2013; Granacher et al., 2016). Less is known on the effectiveness of NT in young athletes. Moreover, findings from NT studies in non-athletic youth cannot directly be translated to young athletes because physiology and proficiency in motor performance differ markedly between non-athletic and athletic populations. Despite the limited knowledge, several national and international scientific organizations recommended to implement NT in young athletes' regular training routines to (i) stimulate their physical and athletic development, (ii) tolerate the demands of long-term training and competition, and (iii) induce long-term health promoting effects that are robust over time and track into adulthood (Behm et al., 2008; Faigenbaum et al., 2016; Lloyd et al., 2016). Therefore, more research is needed on NT-related effects and physiological adaptations in young athletes.

**Figure 1:**
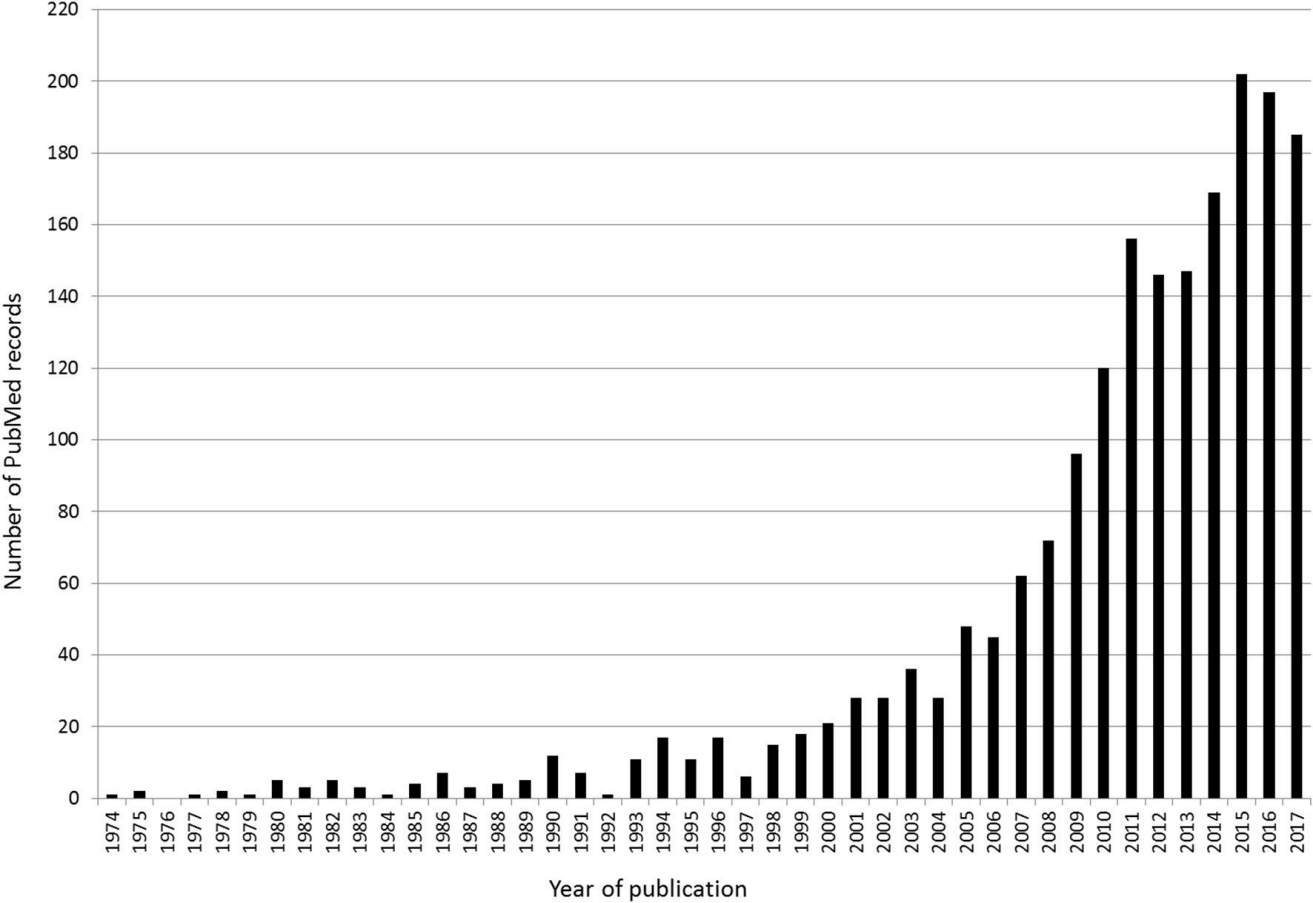
This figure illustrates the results of a systematic search in PubMed according to the following Boolean search syntax: ((“neuromuscular training” OR “strength training” OR “resistance training” OR “plyometric training” OR “power training” OR “stability training” OR “balance training” OR “agility training”) AND (child*OR adolescent* OR youth*)). The following filters were activated: humans; preschool child: 2–5 years; child: 6–12 years; adolescent: 13–18 years. Overall, the search retrieved *N* = 1,999 items.

In 2007, the German Federal Institute of Sport Science (BISp) recognized the discrepancy between these practically relevant but not always scientifically substantiated recommendations (Horn et al., 2012) and launched a new research program with funding opportunities on strength training in young athletes. Consequently, several researchers across Germany intensified their efforts and furthered our knowledge in the field (Behringer et al., 2010, 2011, 2013). As part of the BISp research program, the so-called KINGS-study was established in 2014 which is a 4-year interdisciplinary and multi-centered research project that aims at examining performance-enhancing and health-promoting effects of strength training in young athletes according to sex, maturational status, and sport discipline (https://www.uni-potsdam.de/kraftprojekt/english.php).

Of note, KINGS is an acronym and it stands for the German phrase “**K**RAFTTRAINING **I**M **N**ACHWUCHSLEISTUN**G**S**S**PORT” (engl. Strength Training in Young Athletes). A first achievement of this research consortium was the development and subsequent validation of a conceptual model on the implementation of strength training during the different stages of long-term athlete development (LTAD) (Granacher et al., 2016). Many researchers from the KINGS research consortium acted as authors and editors of this Frontiers Research Topic. We purposely selected the title NT and not just strength training to broaden the scope of the articles that are eligible to be included in this Research Topic. Accordingly, the aims of our Research Topic entitled “Neuromuscular Training and Adaptations in Youth Athletes” were to provide in-depth knowledge in the form of original work, review articles, and meta-analyses on the effects of NT on muscular fitness, athletic performance, and injury prevention in young athletes during the different stages of LTAD.

Overall, 22 articles from 110 authors from Australia, Europe, North and South America were published in this Research Topic. Table 1 outlines a summary of the included articles according to article type, contents, and authors.

**Table 1:** This table contains a summary of the 22 articles published in this research topic entitled “Neuromuscular Training and Adaptations in Youth Athletes” according to article type, contents, and authors.

**Article type**	**Authors**
11 longitudinal studies	Cassel et al.; Granacher and Borde; Hopper et al.; Kobal et al.; Lesinski et al.; Blume et al.; Müller et al.; Prommer et al.; Puta et al.; Ramirez-Campillo et al.; Weber et al.
4 cross-sectional studies	Mersmann et al.; Mueller et al.; Wiewelhove et al.; Prieske et al.
4 systematic reviews and meta-analyses	Behm et al.; Faude et al.; Steib et al.; Gäbler et al.
3 narrative reviews	Mersmann et al.; Bencke et al.; Zemkova et al.
**Contents**	**Authors**
10 articles included child and adolescent athletes	Behm et al; Faude et al.; Mersmann et al.; Müller et al.; Steib et al.; Wiewelhove et al.; Bencke et al.; Gäbler et al.; Weber et al.; Zemkova et al.
10 articles included adolescent athletes	Cassel et al.; Hopper et al.; Kobal et al.; Lesinski et al.; Mersmann et al.; Mueller et al.; Blume et al.; Prieske et al.; Puta et al.; Ramirez-Campillo et al.
2 articles included child athletes	Granacher and Borde; Prommer et al.
15 articles included boys and girls	Behm et al.; Cassel et al.; Faude et al.; Granacher and Borde; Mersmann et al.; Mersmann et al.; Mueller et al.; Müller et al.; Steib et al; Blume et al.; Gäbler et al.; Prommer et al.; Puta et al.; Weber et al.; Zemkova et al.
4 articles included girls	Hopper et al.; Lesinski et al.; Bencke et al.; Prieske et al.
3 articles included boys	Kobal et al.; Wiewelhove et al.; Ramirez-Campillo et al.
11 articles focused on performance-enhancing topics	Behm et al.; Granacher and Borde; Kobal et al.; Lesinski et al.; Müller et al.; Wiewelhove et al.; Gäbler et al.; Prieske et al.; Prommer et al.; Ramirez-Campillo et al.; Zemkova et al.
12 articles focused on health-promoting topics	Cassel et al.; Faude et al.; Hopper et al.; Mersmann et al.; Mersmann et al.; Mueller et al.; Müller et al.; Steib et al.; Bencke et al.; Blume et al.; Puta et al.; Weber et al.
11 articles included physical fitness measures as performance-related outcomes	Behm et al.; Granacher and Borde; Kobal et al.; Lesinski et al.; Müller et al.; Wiewelhove et al.; Gäbler et al.; Prieske et al.; Prommer et al.; Ramirez-Campillo et al.; Zemkova et al.
4 articles included measures of sport-specific or athletic performance	Faude et al.; Müller et al; Gäbler et al.; Zemkova et al.
1 article included measures on cognitive/academic performances	Granacher and Borde
4 articles included lower extremity injury risk factors and rates as health-related outcomes	Hopper et al.; Müller et al.; Steib et al.; Bencke et al.
3 articles focused on tendon overload risk factors and injury rates	Cassel et al.; Mersmann et al.; Mersmann et al.
1 article included sports-related risk factors	Hopper et al.
2 articles included immune status and immunological stress responses	Blume et al.; Puta et al.
1 article focused on low back pain risk factors	Mueller et al.
1 article focused on measures of mental health	Weber et al.
4 articles focused on neuromuscular training	Faude et al.; Hopper et al.; Steib et al.; Zemkova et al.
3 articles focused on strength training	Behm et al.; Puta et al.; Ramirez-Campillo et al.
1 article focused on power training	Behm et al.
2 articles focused on plyometric training	Kobal et al.; Ramirez-Campillo et al.
1 article focused on combined strength and endurance training (concurrent training)	Gäbler et al.
1 article focused on endurance training	Prommer et al.
4 articles focused on sport-specific training	Granacher and Borde; Lesinski et al.; Mersmann et al.; Müller et al.

With regards to number of total views (August, 2018), the top 3 papers of this Research Topic were Behm et al., Steib et al., and Granacher and Borde.

In the form of a systematic review and meta-analysis, Behm et al. examined the effectiveness of traditional strength vs. power training on muscle strength, power and speed with youth. Based on the statistically aggregated findings of 107 studies, moderate effects of power (effect size [ES] = 0.69) and strength training (ES = 0.53) on jump measures. In terms of sprint performances, both power (ES = 0.38) and strength training (ES = 0.48) produced small effects. Finally, power training showed trivial effects on lower body strength (ES = 0.16), while strength training caused large effects (ES = 1.14). More specifically, children and untrained individuals achieved larger ES compared with adolescents and trained individuals. Based on their findings, Behm et al. concluded that strength training should be applied before power training to induce an adequate foundation of strength for subsequent power training activities.

Using a systematic review and meta-analysis, Steib et al. studied the dose-response relationship of NT for injury prevention in youth young athletes. The authors identified 16 trials that examined the effects of NT on lower extremity injuries, including any form of muscular, ligamentous or bony injuries (traumatic or overuse). The authors reported an overall risk reduction of 42% with NT. Training frequencies of 2–3 sessions per week revealed the largest risk reduction, and a weekly training duration of more than 30 min tended to be more effective compared to lower training duration. Finally, interventions lasting more than 6 months were not superior compared with shorter programs.

In an original research article, Granacher and Borde examined the effects of a 1-year sport-specific training and/or physical education on physical fitness, body composition, cognitive and academic performances in young athletes and their non-athletic peers. For this purpose, 45 prepubertal fourth graders from an elite sport class or age-matched peers from a regular class. Young athletes participated in sports that afforded an early start into LTAD (e.g., swimming, gymnastics). Over the 1-year intervention period, the authors observed an average weekly training volume of 620 min for the athletes and 155 min for their non-athletic peers. Sport-specific training did not have a negative impact on growth rates. Better performances were found in physical fitness and physical education grades in favor of the participants from the elite sports class. Similar performances were observed after the intervention for measures of cognition and academics. The authors concluded that sport-specific training in combination with physical education promotes young athletes' physical fitness development during LTAD and does not impede their cognitive and academic performances (Granacher and Borde).

In addition to the above mentioned most frequently viewed papers, another 3 articles from this Frontiers Research topic had a similar scope and focused on muscle and tendon adaptations in young athletes. Mersmann et al. provided a narrative review of current evidence and concepts on the prevention of tendinopathies in young athletes. According to these authors, adolescent athletes are particularly vulnerable to imbalanced development of muscle strength and tendon mechanical properties. This was confirmed in another cross-sectional study of the same research group (Mersmann et al.) in which they provided evidence of imbalanced musculotendinous adaptations in adolescent volleyball athletes compared with age-matched non-athletic peers. These imbalances appear to be a precursor of tendinopathies. There is evidence that these non-uniform musculotendinous adaptations are related to high prevalence rates of tendon overload injuries during maturation (Simpson et al., 2016). Increased levels of circulating sex steroid hormones with growth and maturation could be a critical factor that even augment imbalanced development of muscle strength and tendon mechanical properties (Murray and Clayton, 2013). For instance Cassel et al. showed greater thickness in Achilles and Patellar tendons in adolescent boys compared with girls. Besides growth and sex-related circulating hormones, mechanical loading represents another critical factor that influences the development of muscle and tendon adaptations. In fact, muscle and tendon differ with regards to the time course of adaptation to mechanical loading as well as the responsiveness to certain types of mechanical stimulation. Therefore, it seems that there are tissue-specific (muscle vs. tendon) dose-response relationships that either promote or prevent non-uniform musculotendinous development. For instance plyometric training is characterized by short and intensive bouts of eccentric followed by concentric muscle actions. This stimulus primarily induces neuromuscular but not tendinous adaptations. Consequently, the application of high plyometric training volumes during adolescence may promote the development of musculotendinous imbalances by increasing the risk of sustaining tendon injuries. In their narrative review article, Mersmann et al. provided an evidence-based concept for a specific loading program with the goal to prevent tendon injuries through increased tendon stiffness. This program includes five sets of four repetitions with an intensity of 85–90% of the maximal isometric voluntary contraction and a 3 s movement/contraction duration that provides high magnitude tendon strain (Mersmann et al.).

A rather new and therefore neglected topic in the field of LTAD is how factors like training volume and intensity, performance fatigability, stress and pressure due to school (grades) and competition (success) affect young athletes' mental health. Therefore, Weber et al. studied symptoms of anxiety and depression in young athletes according to age and sex. Overall, 326 young athletes from different sports were enrolled and classified into the age groups late childhood (12–14 years) and late adolescence (15–18 years). Anxiety and depression scores were assessed using the Hospital Anxiety and Depression Scale (HAD Scale). Overall, 7.1% (subclinical scale) and 3.1% (clinical scale) of the young athletes were classified as possible and probable cases suffering from anxiety. In addition, 9.5% (subclinical scale) and 3.7% (clinical scale) of the examined athletes were classified as possible and probable cases for depression. Late childhood athletes showed a slightly lower mean anxiety score compared with late adolescent athletes. No significant age effects were observed for the depression score. Moreover, no sex-related effects were found for anxiety and depression, although female adolescent athletes scored slightly higher in both HAD subscales. The authors concluded that sports medical and sports psychiatric interventional approaches are needed to prevent anxiety and depression in young athletes by teaching coping strategies (Weber et al.).

The 22 articles in this Research Topic furthered our knowledge in the field of NT and adaptations in young athletes. However, there are still voids in the literature. For instance, while Gäbler et al. examined the general effectiveness of concurrent strength and endurance training on physical fitness and athletic performance in youth in the form of a systematic review and meta-analysis, more original research is needed in regards of sequencing effects of strength and endurance training in young athletes. Further, most studies conducted in young athletes focussed on performance-related outcomes following a specific intervention program. The underlying neuromuscular, musculotendinous, and skeletal adaptations are largely unresolved. However, information on physiological mechanisms are crucial to understand maturation and sex-specific dose-response relations. Finally, an important issue not only in elite but also in young athletes is return-to-play (Canty and Nilan, 2015). What are adequate test batteries that can be applied in the laboratory but also in the field during the different stages of rehabilitation to provide information on young athletes' state of recovery? This information is needed to individualize rehabilitation programs and to determine readiness for return-to-play.

## Author contributions

All authors listed have made a substantial, direct and intellectual contribution to the work, and approved it for publication.

### Conflict of interest statement

The authors declare that the research was conducted in the absence of any commercial or financial relationships that could be construed as a potential conflict of interest.
